# The role of schizotypal traits and the *OXTR* gene in theory of mind in schizophrenia: A family-based study

**DOI:** 10.1192/j.eurpsy.2019.17

**Published:** 2020-02-14

**Authors:** M. Giralt-López, S. Miret, J. Soler, S. Campanera, M. Parellada, L. Fañanás, M. Fatjó-Vilas

**Affiliations:** 1 Servei de Psiquiatria, Hospital Universitari Germans Trias i Pujol, Badalona, Spain; 2 Departament de Psiquiatria i Medicina Legal, Universitat Autònoma de Barcelona (UAB), Bellaterra, Spain; 3 Centre de Salut Mental d’Adults de Lleida, Servei de Psiquiatria, Salut Mental i Addiccions, Hospital Universitari Santa Maria, Lleida, Spain; 4 Centro de Investigación Biomédica en Red de Salud Mental (CIBERSAM), Instituto de Salud Carlos III, Madrid, Spain; 5 Departament de Biologia Evolutiva, Ecologia i Ciències Ambientals, Facultat de Biologia, Universitat de Barcelona, Institut de Biomedicina de la Universitat de Barcelona (IBUB), Barcelona, Spain; 6 Departamento de Psiquiatría del Niño y del Adolescente, Hospital General Universitario Gregorio Marañón, Universidad Complutense, Madrid, Spain; 7 FIDMAG Germanes Hospitalàries Research Foundation, Barcelona, Spain

**Keywords:** Family-based study, *OXTR* gene, schizophrenia, schizotypy, theory of mind

## Abstract

**Background.:**

There is consistent evidence that theory of mind (ToM) is impaired in schizophrenia (SZ); however, it remains unclear whether such deficits are trait- or state-dependent. We evaluated ToM in patients with schizophrenia spectrum disorders (SSDs), their healthy first-degree relatives, and controls to test its suitability as an endophenotypic marker. We also studied the modifying effect of markers of clinical and genetic liability to SZ (schizotypy and genetic variability in the oxytocin receptor gene: *OXTR*) on ToM in healthy individuals.

**Methods.:**

The sample included 38 stable SSD patients, 80 unaffected first-degree relatives, and 81 controls. ToM was assessed using the Hinting Task (HT) and schizotypy via the Schizotypal Personality Questionnaire-Brief (SPQ-B), which generates interpersonal (SPQ-IP), cognitive-perceptual (SPQ-CP), and disorganization (SPQ-D) scores. The polymorphism rs53576 of *OXTR* was genotyped.

**Results.:**

Patients presented poorer HT performance than relatives and controls (*p* = 0.003 and *p* < 0.001). High SPQ-IP and SPQ-CP scores correlated with poorer ToM performance in relatives (*p* = 0.010 and *p* = 0.030), but not in controls. *OXTR* was not associated with HT scores, but it showed a modifying effect within controls; high SPQ-CP was related to HT poorer performance conditional to GG genotype (*p* = 0.007).

**Conclusions.:**

ToM deficits were present in patients but not in unaffected relatives or controls. However, our data indicate the usefulness of clinical and genetic liability markers to characterize differences in ToM abilities within healthy individuals. Then, the observed link between ToM and SZ liability suggests the putative role of ToM as an endophenotypic marker. Nevertheless, new analyses in larger samples are needed.

## Introduction

Schizophrenia (SZ) is a prevalent and severe psychiatric disorder with a complex etiology involving environmental and genetic factors (heritability ≅ 80%). The high degree of disability, prevalence, chronicity, and financial costs place an enormous burden on patients with SZ, their families, and society as a whole. This burden warrants efforts to identify predictors directly applicable to prevention, diagnosis, and therapy. Research into these predictors has produced growing evidence that social cognition deficits are an important predictor of outcome, even more so than neurocognition [1]. Social cognition refers to a wide range of skills that allow people to perceive, interpret and process social stimuli, and that guide social interactions. One of its multiple components is theory of mind (ToM), that is, the ability to infer mental states, such as beliefs, intentions, desires, and emotions, in other people [2].

ToM impairments are being increasingly reported in SZ [3–5]. Although some studies have interpreted ToM deficits as a state marker associated with symptoms in the active phase of the disease [6] or with the severity of negative symptoms [7], the majority have reported ToM impairment to be a trait marker, still present in remission [8,9] and found to be stable after a longitudinal 3-year follow-up design [10]. Several studies also indicate that ToM deficits are present in first-episode psychosis and in high-risk individuals (unmedicated prodromal subjects) [4,11].

Family-based study designs are suitable for establishing whether ToM is a trait feature when the defect is also seen in healthy family members (in a higher prevalence than in controls). Although large samples of well-characterized families are difficult to recruit, these studies have the advantage of controlling for certain confounder factors, as family members have a shared genetic background and tend to be more homogeneous with respect to exposure to environmental factors.

Meta-analytic data on social cognition in unaffected relatives of patients with SZ have reported their decreased performance when compared to healthy controls [12] and that the social cognition abilities  in relatives lie somewhere between the levels seen in probands and in healthy non-relatives [4].

All of the above, in addition to the fact that some social cognitive impairment are heritable (28–37% for emotion recognition) [13], account for the interest in social cognition as a candidate endophenotype.

To explain the heterogeneous results in the relative group and to detect healthy relatives with a potentially higher genetic loading for SZ, a useful strategy would be to study the association between the endophenotypic marker and other known vulnerability markers in SZ such as schizotypy, family history, and specific genetic factors. Schizotypy is a set of personality traits that encompasses behaviors, cognitions, and emotions, and resembles the signs and symptoms of SZ in the general population. It has been associated with ToM impairment [14,15].

As regards the biological underpinnings of ToM, the most extensively studied neuropeptide to date is oxytocin (OXT). On the one hand, several functional neuroimaging studies have shown that OXT has significant modulatory effects on “social brain,” which refers to the brain systems that govern social cognition, social behavior, and affect regulation [16]. For example, acute OXT administration in healthy subjects has been shown to modulate amygdala activity as well as other regions (e.g., prefrontal areas, superior temporal sulcus, and fusiform gyrus) and to enhance the functional connectivity of the amygdala with other brain regions [17,18]. Also, animal models of psychosis have shown that OXT administration reduces dopaminergic hyperactivity in the striatum and nucleus accumbens [19] in a similar manner to antipsychotic medications [20,21].

On the other hand, some studies in healthy subjects have shown the enhancing effect of intranasal OXT administration on social cognition [22] and specifically on ToM [23] and that this is an age-independent effect [24]. A meta-analysis based on neurodevelopmental disorders (SZ and autism spectrum disorders) also reported the age-independent intranasal OXT improvement effect on ToM across these disorders [23]. Accordingly, it has been suggested that both the anatomy and functional physiology of the human nervous system may be shaped by OXT signaling pathways (and their interaction with dopaminergic ones), leading to the idea that their variability may contribute to individual differences in social behavior and cognition by modulating the neural circuits involved in processing socio-affective information [25,26].

Importantly, the behavioral effects of OXT depend on the distribution and expression of its receptor (*OXTR*). OXT receptors are present in numerous limbic and reward-related regions of the human brain; mainly in (but not restricted to) the amygdala, the hippocampus and the nucleus accumbens, and also in cortical regions (such as the anterior cingulate cortex) [27]. The receptor is coded by the *OXTR* gene and its polymorphic variability may contribute to individual differences in social behavior and cognition [25].

General population-based studies have highlighted the influence of this gene on many facets of social cognition including ToM. The most intensively examined single nucleotide polymorphism (SNP) in *OXTR* is rs53576, and a recent meta-analysis showed that individuals with a greater number of G alleles present better empathic ability [28]. Interestingly, Rodrigues et al. [29] linked the lower empathy exhibited by A allele carriers of this SNP with a higher physiological stress reactivity as compared to GG individuals.

Furthermore, *OXTR* polymorphic variants (rs53576 and others) have been associated with the risk for SZ and with other neurodevelopmental disorders (reviewed in [17]).

Neuroimaging studies have provided evidence to support the rs53576 association with brain structure and activity, mainly in healthy subjects. For instance, Tost et al. [30] described that A allele carriers of rs53576 show the lowest amygdala activation and an increased coupling of the hypothalamus and the amygdala when processing social information. Data also suggest that rs53576 influences striatal dopamine availability and modulates the interactions between the oxytocinergic and dopaminergic systems. In a study based on single-photon emission-computed tomography (SPECT), striatal dopamine transporter availability in G carriers (AG/GG) was lower than in the AA group, and G carriers showed a negative correlation between dopamine transporter availability and OXT level [31]. Then, despite rs53576 is a silent polymorphism and that the pathophysiological significance of its association with brain phenotypes remains to be elucidated, accumulated evidence suggest that this SNP could be a marker of the role of the *OXTR* in the neural mechanism that links the differences in the oxytocinergic system to individual differences in social cognition.

In line with the above, our study first aimed to explore ToM in schizophrenia-spectrum disorder (SSD) patients, healthy first-degree relatives, and healthy controls to test whether ToM deficits may be a putative endophenotypic marker of SZ. We hypothesized that ToM deficits would show a trait marker pattern with first-degree relatives showing intermediate scores between patients and healthy controls. Second, to understand the ToM variability, we aimed to investigate whether ToM is modified by: (i) clinical liability to SZ in healthy individuals (schizotypy) and (ii) the OXT receptor gene (*OXTR*)*.* We hypothesized that the polymorphism rs53576 at the *OXTR* would modify the association between schizotypy and ToM, and that the allelic variants would co-segregate with ToM performance within families.

## Methods

### Sample

The sample comprised 199 individuals: 38 patients with a diagnosis of a SSD, 80 healthy first-degree relatives of these patients (22 fathers, 30 mothers, and 28 siblings), and 81 unrelated controls with no psychiatric history (Table 1). All participants were of Caucasian origin. All were recruited at the Centre de Salut Mental d’Adults de Lleida and evaluated by the same clinician (S.M.), and were assessed when they were clinically stable.

**Table 1.** Sample description and statistical comparisons among patients, first-degree relatives, and controls

	Patients (*n* = 38)	First-degree relatives (*n* = 80)	Controls (*n* = 81)	ANOVA *F*/*χ* ^2^ (*p*)	Post hoc significant differences	
Male (%)	73.68	41.25	45.68	11.54 (0.003)	P > C, P > R	
Age at interview	24.92 (3.90)	44.74 (13.43)	34.77 (12.53)	38.46 (<0.001)	R > C > P	
Years of education	13.43 (3.12)	11.42 (4.83)	15.23 (3.82)	16.59 (<0.001)	C > R, P > R	
IQ	90.03 (15.23)	94.97 (15.26)	107.98 (11.96)	27.67 (<0.001)	C > R, C > P	
PANSS positive	10.63 (3.36)	–	–			
PANSS negative	20.37 (4.01)	–	–			
PANSS general	32.58 (7.32)	–	–			
PANSS “lack of insight”	2.50 (0.80)	–	–			

Proportion (%) or mean scores (SD). Only significant differences in post hoc comparisons are given (all *p*-values were <0.001).

Abbreviations: C, controls; P, patients; R, first-degree relatives.

Patients’ DSM-IV-TR diagnoses were: SZ (*n* = 32) and psychotic disorder not otherwise specified (*n* = 6). Patients’ mean age at onset was 22.12 (SD = 3.83) and the mean duration of illness was 26.40 months (SD = 24.44).

All patients were treated with antipsychotic monotherapy: 94.7% with second-generation antipsychotic (24 risperidone, 5 olanzapine, 3 amisulpride, 2 ziprasidone, 2 clozapine) and 5.3% with haloperidol.

The exclusion criteria for relatives included any psychotic spectrum disorder and any major affective disorder. Controls had no personal or family history of psychiatric disorders or treatment.

Other exclusion criteria common to all groups were any major medical illness that could affect brain function, neurological conditions, and history of head trauma with loss of consciousness.

All participants provided written consent after being informed of the study procedures and implications. The study was performed in accordance with the guidelines of the institutions involved and approved by the local research ethics committees. All procedures were carried out in accordance with the latest version of the Declaration of Helsinki.

### Assessments

The patients were diagnosed according to DSM-IV-TR criteria and interviewed by means of the Comprehensive Assessment of Symptoms and History [32].

Symptom severity and prevalence of positive or negative symptoms were assessed by means of The Positive and Negative Syndrome Scale (PANSS) [33]. It is a 30-item scale designed to obtain a measure of positive, negative, and general psychopathology symptoms in patients with SZ. The scores for these scales are arrived at by summation of ratings across component items. In addition to these measures, a Composite Scale is scored by subtracting the negative score from the positive score. This yields a bipolar index, which is essentially a difference score reflecting the degree of predominance of one syndrome (positive or negative) in relation to the other.

Based on the previously reported association between insight and social cognition [34], the PANSS item “lack of insight” was also independently used to test such association.

Social cognition, specifically ToM, was assessed by means of the Spanish version of the Hinting Task (HT) [35,36], a test that consists of 10 brief stories involving two people in a conversation. The task is to infer what a person is implying indirectly. In each item, a correct answer gives 2 points (for a total of 20 points). In case of an incorrect answer, an additional hint is given, after which a correct answer gives 1 point.

The task has good validity for patients with SZ and has proven sensitive to ToM difficulties in a number of studies to date [37].

Schizotypy assessment was carried out by means of the Schizotypal Personality Questionnaire-Brief (SPQ-B) [38]. SPQ-B is a 22-item self-report; each item presents a statement or question to the respondent, who then circles “yes” or “no.” Each affirmative response counts as one point toward the total score, which ranges from 0 to 22, with higher scores indicating higher levels of self-reported schizotypy. Items were created to measure three schizotypal dimensions: cognitive-perceptual dimension (i.e., ideas of reference or odd beliefs), interpersonal dimension (i.e., suspiciousness, inappropriate, or constricted affect), and disorganization dimension (i.e., odd thinking/speech/behavior/appearance). In order to select a subgroup of carriers of higher-vulnerability SPQ subscales, raw scores were dichotomized using the SPSS visual binning method in each group to define high/low scorers for each SPQ subscale.

Intellectual quotient (IQ) was estimated using the Block Design and Vocabulary or Information WAIS-III subtests.

Family history was assessed with the Family Interview for Genetic Studies. Following broad SZ spectrum criteria [39], families were classified as having a positive family history when patients had at least one first- or second-degree relative with SZ, affective or nonaffective psychosis, or schizotypal or paranoid personality disorder.

### Molecular analysis

Genomic DNA was extracted from peripheral blood cells or buccal mucosa using standard methods, that is, the Real Extraction DNA Kit (Durviz S.L.U., Valencia, Spain) or the BuccalAmp DNA Extraction Kit (Epicenter Biotechnologies, Madison, WI).

The *OXTR* gene on chromosome 3p25 spans ~19 kbp and contains four exons and three introns. Genotyping of the intronic SNP rs53576 in the *OXTR* gene was performed using a fluorescence-based allelic discrimination procedure (TaqMan 5′ exonuclease assays; Applied Biosystems). Standard conditions were observed. The genotyping call rate was higher than 86%. After randomly regenotyping 10% of the sample, 100% of the genotyping results were confirmed.

The SNP was in Hardy–Weinberg equilibrium. Due to the low frequency of individuals homozygous for the allele A, the genotype variable was dichotomized in GG versus A allele carriers (AA+AG).

### Statistical analyses

All data were processed using SPSS 22.0 software (SPSS IBM, Armonk, NY).

Sociodemographic and clinical data were compared between groups by means of analysis of variance (ANOVA) tests or a chi-squared test when appropriate.

The effect of age, years spent in education, and IQ on HT performance for each group was tested using the Pearson correlation test. Within groups, differences on HT performance according to sex or family history were tested using Student’s *t*-test. From all these analyses, age and sex showed a trend toward or significant effect in relatives (*p* = 0.059 and *p* = 0.001, respectively), so they were added as covariates in the subsequent analyses.

The association between HT performance and clinical characteristics (PANSS) within patients was tested with linear regressions (adjusted for age and sex).

HT performance differences between patients and their first-degree relatives were assessed by means of linear mixed models (LMMs) with total HT as the dependent variable, family member (patients/first-degree relative) as the fixed-effect factor, sex and age as fixed-effect covariates, and family as the random effect (subjects nested within families). When the analyses included nonrelated groups (patients vs. controls and relatives vs*.* controls), the same models were used for the comparison without including the family random effect.

The association between HT performance and schizotypy within healthy relatives and controls was tested with linear regression (adjusted for age, sex, and family history).

To test the putative effect of the *OXTR* gene as a mediator of the relationship between HT performance and schizotypy, the genotype (GG vs. A allele carriers) was added to these analyses.

In addition, a family-based association test between rs53576 and the HT scores was conducted with PLINK v1.07 by means of the quantitative transmission disequilibrium test (qTDT). Since PLINK does not allow covariates to be included in qTDT analyses, the analyses were performed in two steps. First, lineal regressions between HT scores and each schizotypy factor (covaried by age and sex) were conducted. Second, the residuals from these regressions were used to conduct the qTDT.

## Results

### Sample characteristics

Tables 1 and 2 show the main sociodemographic and clinical data of the sample. The proportion of males was higher in the patients group (73.7% male) than in the relatives (41.3%) or controls (45.7%) groups. Relatives had spent less time in education than patients or controls. Such a difference was mainly attributable to parents (mean years of education [SD] = 9.46 (4.05) and not to siblings (mean years of education [SD] = 14.93 (4.12)]. IQs were higher in the control sample than in the relatives or patients. A total of 21.1% of patients and 17.5% of relatives had a family history of psychotic disorders.

**Table 2.** ToM and schizotypy (SPQ-B) scores in patients (P), their first-degree relatives (R), and controls (C)

HT^a^	Patients		First-degree relatives		Controls	
	15.74 (4.07)		17.88 (2.16)		18.46 (1.64)	
	Low scorers	High scorers	Low scorers	High scorers	Low scorers	High scorers
SPQ-CP^b^	0.91 (0.81)	4.15 (1.41)	0 (0)	2.00 (1.11)	0 (0)	2.00 (1.11)
SPQ-IP^b^	2.38 (1.50)	6.55 (1.13)	1.60 (1.01)	4.96 (1.19)	1.05 (0.83)	4.03 (1.15)
SPQ-D^b^	0 (0)	2.24 (1.48)	0 (0)	1.63 (0.92)	0 (0)	1.39 (0.72)

Schizotypy scores for each dimension were dichotomized in low and high scorers. Mean scores (SD).

Abbreviations: HT, Hinting Task; SPQ-CP, Schizotypal Personality Questionnaire-cognitive-perceptual; SPQ-D, Schizotypal Personality Questionnaire-disorganization; SPQ-IP, Schizotypal Personality Questionnaire-interpersonal.

a Available for 39 patients, 82 relatives, and 81 controls.

b Available for 36 patients, 76 relatives, and 68 controls.

Within the family group, there were no significant differences in HT or SPQ-B scores between siblings and parents (data not shown); for this reason, they were considered a single group in all analyses.

According to the PANSS Composite Scale, all patients showed more prevalent negative than positive symptoms. When we tested whether ToM performance in patients is modulated by clinical severity or insight as measured with PANSS, no association was detected. Thus, these clinical variables were not considered in the following analyses.

### Analysis of ToM performance in SSD patients relative to their first-degree relatives and healthy individuals

A comparison of HT performance between patients and first-degree relatives and between patients and controls showed significant group effects (*F* = 8.96, *p* = 0.003 and *F* = 17.64, *p* < 0.001, respectively). Patients presented lower scores than relatives (estimated mean difference = −2.27) and controls (estimated mean difference = −2.44). These results remained significant after including IQ as a covariate (*p* = 0.007 and *p* = 0.025, respectively). The scores of relatives and controls did not differ significantly.

### Association between HT performance and schizotypy in healthy relatives and controls

We tested whether ToM performance in healthy individuals is modulated by schizotypy, a marker of liability to SSD.

A linear regression analysis (adjusted for age, sex, and family history) showed that being a high scorer for Schizotypal Personality Questionnaire-interpersonal (SPQ-IP; *β* = −0.277, *p* = 0.010, *R*_adj_^2^ = 22.2%) and Schizotypal Personality Questionnaire cognitive-perceptual (SPQ-CP; *β* = −0.243, *p* = 0.030, *R*_adj_^2^ = 20%) was related to poorer ToM performance in relatives. Results for SPQ-IP remained significant when the IQ was included as a covariate (*p* = 0.025). No such relationship was observed in the controls.

### Association between OXTR gene (rs53576) and ToM performance

For the *OXTR* SNP, the genotype distribution is shown in Table 3.

**Table 3.** Genotype frequencies for the single nucleotide polymorphism rs53576 at the *OXTR* gene and HT mean scores within each group

	Patients (*n* = 37)			Relatives (*n* = 76)			Controls (*n* = 56)		
	GG	GA	AA	GG	GA	AA	GG	GA	AA
Genotype frequencies	21 (56.8%)	15 (40.5%)	1 (2.7%)	43 (56.6%)	31 (40.8%)	2 (2.6%)	31 (55.4%)	21 (37.5%)	4 (7.1%)
HT mean score (SD)	15.24 (4.83)	16.20 (3.03)	16.00	17.77 (2.40)	17.97 (1.96)	19.50 (0.71)	18.58 (1.61)	18.38 (1.80)	18.00 (1.63)

The qTDT analysis showed no association between the *OXTR* gene and HT performance within families.

The rs53576 (GG vs. A allele carriers) did not show a significant association with HT performance but showed a modifying effect on the relationship between schizotypy and HT in controls. When including the *OXTR* variability in the model (see “Association between Hinting Task performance and schizotypy in healthy relatives and controls” section) being a high scorer for SPQ-CP turned to be related to poorer ToM performance in controls (*β* = −0.307, *p* = 0.030, *R*_adj_^2^ = 13.8%). Accordingly, within GG subjects (17 SPQ-CP low scorers and 12 high scorers), the effect of SPQ-CP on HT performance was statistically significant (*β* = −0.468, *p* = 0.007, *R*_adj_^2^ = 30.1%), while it was not within A carriers.

In relatives, the association between SPQ and HT remained significant while the genotype did not show an effect per se.

## Discussion

In our study, SZ patients showed impairments in inferring the mental states of others (i.e., intentions) via indirect speech such as hints, measured with the HT, in comparison to controls. This result is consistent with previous studies that have used the same task [40–42] and others that have used different tools for assessing ToM [3,5,8,11].

To further investigate the properties of ToM as an endophenotypic marker, we looked at how healthy relatives behave in relation to patients and controls. In our study, the mean score of healthy relatives in the HT fell somewhere between the scores of the other two groups, but the differences between relatives and controls were not significant. In line with our findings, other studies that have assessed ToM aspects have also observed no differences in relatives compared to controls [43,44]. In contrast, other studies have suggested a genetic liability effect with relatives demonstrating impairments in advanced ToM ability [4,45,46]. A recent study that included several different measurements reported mixed results across tasks [9].

This heterogeneity of results could be explained by different factors including the sample sizes of studies and the different tasks used to evaluate ToM.

First-order false belief or deception tasks test the ability to understand that someone can have an inaccurate mental representation of events based on incomplete knowledge. In a second-order false belief or deception task, participants have to infer the (false) beliefs of one character about the (false) beliefs of a second character [47,48].

More complex ToM includes the comprehension of indirect speech, such as irony, metaphors, faux pas, and hints, assessed using the Strange Stories Task [49] or the HT. This is based on the notion that understanding indirect speech requires an understanding of another person’s mental state.

It has been reported that ToM abilities might decline at different times over the course of SZ. This is consistent with Brüne’s developmental model, which posits that ToM abilities decline in the reverse order of acquisition [5]. Therefore, deterioration is first detected in complex ToM tasks, while the decline in first-order ToM tasks is observed later. This theory is consistent with studies that have observed a deficit in second-order mentalizing questions, but not in first-order inference items, in patients experiencing a first episode of psychosis [50]. Thus, it can be deduced that tasks assessing higher-order forms of ToM could be more sensitive and especially appropriate for detecting ToM impairment in nonclinical samples such as healthy relatives. Also, there could be other factors intrinsic to the task that conditions its sensitivity to changes in social cognition abilities performance. For instance, Grainger et al. [24] detected that intranasal OXT administration improved ToM when the task had minimal contextual information, but not when the task had enriched contextual information.

Another element to consider when explaining the heterogeneity of ToM capacities in the healthy relatives’ group is the variability in clinically defined liability. As regards to the selection of schizotypy, it is a clinical liability marker that has shown to be useful in the identification of families with a higher genetic loading for SZ [51]. In this respect, our study shows that high levels of positive and negative schizotypy (cognitive-perceptual and interpersonal dimensions) are related to poorer ToM performance in relatives but not in controls. Within the general population, ToM has been extensively studied in relation to schizotypy. Several studies have detected ToM impairments associated with high levels of total schizotypy [14,15]. In line with our results, other studies have failed to identify this association in controls [52,53], but some have reported that poorer social cognition is a function of specific schizotypy traits such as positive schizotypy [54,55].

Overall, the mixed findings associated with schizotypy and ToM performance in healthy controls may be due to the fact that individuals with positive and negative schizotypy display poor ToM performances for different reasons. Frith [56] asserted that individuals with positive symptoms of SZ show deficits in ToM tasks due to inaccurate representations of the intentions of others, whereas individuals with negative symptoms of SZ display ToM deficits due to a lack of experience of or interest in social interactions. It may also be that different aspects of social cognition are differentially affected by positive and negative schizotypy.

Another fact that could explain the heterogeneity is the different ways of quantifying schizotypy levels. Some studies have used a continuous variable, while others have used median splits or the top and bottom 5 or 10% in an attempt to emphasize the qualitative differences between groups. We were not able to use this method due to the limited size of our sample.

In contrast to the results for the control group, data from the subgroup of healthy relatives of SZ-spectrum patients suggest that high levels of cognitive-perceptual and interpersonal dimensions are related to poorer ToM performance. To the best of our knowledge, only three studies have tested this association in a sample of healthy relatives of SZ patients. Two found no significant correlations between ToM and schizotypy scores in the relative sample [57,58]. However, SPQ mean scores and ranges in these samples were somewhat low.

Irani et al. [59] used the Revised Eyes Test and reported a trend whereby relatives were more accurate than patients and less accurate than controls in the ToM task, although these differences only became significant when the SPQ social-interpersonal subscale scores were included. This is consistent with the hypothesis that these social-interpersonal features are the best differentiators between relatives and controls, and might be the most important schizotypal traits associated with genetic vulnerability for SZ [60]. Despite the fact that the relatives in our sample showed higher schizotypy levels than controls on the social-interpersonal subscale, differences were not significant.

In our study, schizotypy symptoms in healthy first-degree relatives seem to put individuals at an increased risk of these ToM deficits, thus suggesting that some social cognition functions might be sensitive to subthreshold psychotic symptoms. These results are similar to those reported by Johnson et al. [61], in which the relationship between schizotypy and other measures of cognition was mediated by SZ genetic risk.

Finally, regarding the role of the *OXTR* gene in ToM, our study did not report a family-based association. However, despite not having an effect per se on HT, the genotype showed a modifying effect of the association between schizotypy and HT in controls. Then, while the genotype did not change the significant relationship observed between schizotypy and HT within relatives, in controls, this association became significant when the polymorphism was included. This suggests that in controls (with less liability load than relatives) high schizotypy would be related to a poorer performance on ToM conditional to GG genotype. This result is consistent with other studies that observed the role of this polymorphism on different social cognition domains in healthy subjects [25–27], nonetheless the comparison is not straightforward as previous studies were based on different social cognition dimensions or scales and none has analyzed the role of *OXTR* gene jointly with schizotypy. In all, these results should be treated with caution, as the sample size limits the statistical power of our study and replication is needed in larger samples in order to confirm such effect.

Given the well-documented role of OXT in mammalian social behavior, and the previously mentioned effects of OXT administration on social abilities in humans, it is not surprising that *OXTR* gene variability has been studied with a range of social phenotypes [62]. In this regard, some studies have provided evidence that *OXTR* polymorphisms are associated with different dimensions of social cognition in healthy individuals [63,64] and also with the risk for SZ and poorer performance in ToM measurements in SZ, while others have failed to find such an association [17,65,66].

Focusing on the family-based approach, despite the design being particularly robust for controlling widespread confounds such as admixture and stratification [67], we are aware of only one previous study with a similar approach as ours. In that study, Wade et al. [67] reported the association of another polymorphism at intron 3 of *OXTR* (rs11131149) with social cognition in 18 months old children. Interestingly, the same authors also reported the interaction between *OXTR* and parenting behavior on 4 years old children’s ToM [68], suggesting a nature–nurture interaction with regard to ToM in early development. Therefore, our results add to the variability and discrepancies observed in the limited body of literature in relation to SZ, which are likely related to several factors such as the diversity of genotypes across studies, the variation in participants’ ancestry and limited sample sizes. Also, for the understanding of heterogeneity across studies, our data and other family-based studies indicate the need of considering environmental and developmental factors.

To interpret our data, it is important to consider the strengths and limitations of the study. The strengths include the use of a family-based design with a control group of healthy subjects and the availability of social cognition measurements and clinical and genetic liability markers. In addition, only a few studies have explored the association between social cognition and schizotypy, and none of these has incorporated data on genetic liability (family history and genetic variability). The design of our study and the phenotypic variability therefore contributes to the state–trait debate and helps shed light on the heterogeneity of the complex traits related to SZ liability.

On the other hand, even though the sample size is comparable to previous studies and that one of the main disadvantages of family-based designs is the challenge associated with recruiting large samples of well-characterized families, the size of our sample represents the main limitation of the study. In line with this, the family-based design is intrinsically associated with differences in sample sizes of the subgroups (more relatives than patients), which could represent a bias when conducting subgroups comparison analyses. Moreover, although ToM is an important area of social cognition, other areas, such as emotion processing and social knowledge, were not studied. Also, according to some recent data showing that antipsychotic treatment can improve social cognition performance [69,70], the non-inclusion of the treatment data in our analyses could limit the interpretation of our results. Then, if we had excluded the treatment effect, the patients’ performance on ToM could be potentially worse and could show larger differences with relatives and healthy controls. Finally, although one of the most studied polymorphisms in the *OXTR* gene is rs53576, the analysis of a single SNP does not represent the whole variability gamut of *OXTR.* In addition, like many human competencies, ToM is a complex trait influenced by multiple genes, future research should screen the polymorphic variability along the *OXTR* gene and other OXT signaling pathway-related genes.

In conclusion, our study initially shows that ToM deficits are greater in patients with SSDs as compared to healthy relatives and controls; however, when clinical and genetic  liability markers such as schizotypy and *OXTR* gene in healthy subjects are considered, our data indicate the putative role of ToM as a trait marker. Our study does not report the role of the polymorphism rs53576 at the *OXTR* gene on ToM abilities within families; however, new studies in larger family-based samples are needed.
